# Dataset to support the modelling of Vietnam’s transport sector: population, economy, transport activity, energy intensity, load capacity, and carbon emission

**DOI:** 10.1016/j.dib.2026.112898

**Published:** 2026-05-29

**Authors:** Naomi Tan, Fynn Kiley, Holger Dalkmann, Johanna Zilliacus, John Harrison, Mark Howells, Vivien Foster

**Affiliations:** a Centre for Sustainable Transitions: Energy, Environment and Resilience, Loughborough University, Loughborough LE11 3TU, United Kingdom; b Centre for Environmental Policy, Imperial College London, London SW7 1NE, United Kingdom; c Sustain 2030, Berlin, Germany; d Consultant, Asian Development Bank, Mandaluyong City 1550, Metro Manila, Philippines

**Keywords:** MAED, OSeMOSYS, Passenger activity, Freight activity, Energy efficiency, Transport emissions

## Abstract

The rapid growth of Vietnam’s transport sector presents challenges for sustainable energy and transport planning, particularly due to rising fuel consumption and associated carbon emissions. To help address these challenges, this paper presents a comprehensive dataset designed to support the modelling of future transport demand, energy use, and CO_2_ emissions in Vietnam. The dataset covers population, GDP, passenger and freight activity, vehicle stock, energy intensity, load capacity, and CO_2_ factors across nine transport modes: motorcycles, cars, buses, light-duty vehicles, heavy-duty vehicles, rail, inland waterways, maritime, and aviation. These are further disaggregated into nine fuel types: petrol, diesel, compressed natural gas, electricity, biofuel, fuel oil, hydrogen, ammonia, and jet fuel. Data were compiled from national statistics, government reports, online databases, academic journals, and media sources. Structured for use with open-source modelling tools, the dataset supports analyses of transport demand and carbon accounting, offering researchers, policymakers, and consultants a resource to evaluate long-term decarbonisation pathways and inform evidence-based policymaking.

Specifications Table

**Table:** 

Subject	Earth & Environmental Sciences
Specific subject area	Transport demand and emissions modelling
Type of data	Table, Chart, Graph. Raw, analysed, processed.
Data collection	The dataset is compiled from open-access sources to ensure transparency and replicability. Where data were unavailable, estimates were derived through calculations or proportional assumptions based on reliable sources to represent Vietnam’s transport landscape comprehensively. Sources for the dataset include national statistics, government reports, online databases, academic journals, and media outlets.
Data source location	The sources of the raw data are listed in the repository and references section. The location of this dataset can be found at: https://zenodo.org/records/17093877
Data accessibility	Repository name: Zenodo Data identification number: 10.5281/zenodo.17093877 Direct URL to data: https://zenodo.org/records/17093877
Related research article	None

## 1 Value of the Data

• The dataset disaggregates Vietnam’s transport sector by mode and fuel type, advancing existing data [[1], [2], [3], [4]], which are typically reported at an aggregated modal level. This enables policymakers and planners to assess recent trends from 2015 to 2024, evaluate policy impacts, and develop detailed evidence-based transport and energy transition strategies under different economic and technological pathways [[1], [2], [3], [4]].
• The dataset offers a consistent and transparent foundation for scenario analysis, and is designed for compatibility with open-source energy modelling tools, allowing modelling practitioners and professionals direct uptake in transport demand and energy system models without extensive data pre-processing.
• The methodologies used to develop the dataset are explicitly documented and replicable, allowing researchers to adopt or adapt the approach to transport data development in other countries with similar data constraints. For example, the methodologies can be applied to more aggregated foundational datasets available for other countries [1] to derive consistent mode-fuel level data for detailed transport modelling.
• The dataset and its documentation provide a practical example of transport data harmonisation and uncertainty management, making it suitable for energy data and modelling capacity building in academic and professional training contexts.
• The dataset includes thorough metadata, including specific links and sources, the Statistical Data and Metadata eXchange (SDMX) observation status for each entry, and a data traffic-light system to enhance transparency and user confidence in data quality and uncertainty handling. This is an advancement over prior datasets by embedding data quality assessment directly within the dataset structure, rather than requiring users to independently evaluate data reliability.

## 2 Background

Vietnam's transport sector is rapidly expanding, with passenger and freight activity increasing more than fourfold over the past two decades [1], driven by fast economic growth and urbanisation [5,6]. Thus, to support Vietnam’s net-zero carbon plan announced at COP26, structured and accessible data are needed to inform low-carbon transport pathways. This dataset fills this gap and supports the modelling and analysis of Vietnam’s transport sector. Following the database structure of Tan et al. [1], data from 2015 to 2024 is compiled to capture recent trends in economic development and policy impacts. The dataset is disaggregated by transport mode and fuel type and includes emission factors for carbon dioxide (CO_2_). The dataset is compiled from open-access sources to ensure transparency and replicability. Where data is unavailable, estimates are derived through calculations or assumptions based on reliable sources, such as the Vietnam National Statistics Office (NSO). Other sources for the dataset include national statistics, government reports, online databases, academic journals, and media outlets. Designed for integration into energy modelling tools, the dataset consists of detailed metadata, including links, the Statistical Data and Metadata eXchange (SDMX) observation statuses, and a traffic-light quality system, providing a robust basis for scenario-based analysis and data-driven policymaking.

## 3 Data Description

The dataset, as noted in Table 1, includes population, GDP, passenger activity, freight activity, vehicle stock, energy intensity, load capacity, and CO_2_ emission factors for nine vehicle types across five transport sectors: motorcycle, car, bus, light-duty vehicle (LDV), heavy-duty vehicle (HDV), rail, inland waterway (IWW), maritime, and aviation. Both international and domestic transport are included for maritime and aviation. Different fuel types were also considered, including petrol, diesel, compressed natural gas (CNG), electricity, biofuel, fuel oil (such as heavy fuel oil or bunker fuel), hydrogen, ammonia, and jet fuel, as summarised in Table 2. Non-motorised modes, such as walking and cycling, are excluded from the dataset. The transport modes were selected and defined following the 2018 Vietnam Transport and Logistics Statistical Yearbook (TLSY) [2], with minor adjustments. They are summarised as follows:

• Motorcycle: two- or three-wheeled motor vehicles, including mopeds
• Car: vehicles with up to nine seats, including the driver
• Bus: vehicles with 10 or more seats
• LDV: cargo vehicles with a maximum capacity of 2 tonnes
• HDV: cargo vehicles with a capacity exceeding 2 tonnes
• Rail: railway cars transporting passengers and/or goods. This does not include baggage and dining coaches.
• IWW: boats or vessels operating on inland waterways, with or without engines
• Maritime: vessels used for maritime transport operations. This does not include service structures or pleasure yachts.
• Air: aeroplanes, excluding specialised aircraft such as helicopters and seaplanes

**Table 1:** A summary of the data and its corresponding variables, time horizons, and units.

Data	Variable	Time horizon	Unit
Population	Total population	2015–2050	Million people
	Population growth	2015–2050	%
GDP	Total GDP	2015–2050	Billion USD (2015)
	GDP growth	2015–2050	%
Passenger activity	Passenger (see Table 2)	2015–2024	Million passenger-km
Freight activity	Freight (see Table 2)	2015–2024	Million tonne-km
Vehicle stock	Passenger and freight combined (see Table 2)	2015–2024	Units
Energy intensity	Passenger (see Table 2)	Time-independent	MJ/passenger-km
	Freight (see Table 2)	Time-independent	MJ/tonne-km
Load capacity	Passenger (see Table 2)	Time-independent	Passenger/vehicle
	Freight (see Table 2)	Time-independent	Tonne/vehicle
CO_2_ emission factor	Passenger (see Table 2)	Time-independent	gCO_2_/passenger-km
	Freight (see Table 2)	Time-independent	gCO_2_/tonne-km

**Table 2:** The transport sectors, vehicle types, and fuel types defined in the dataset.

	Road					Rail	IWW	Maritime*	Air*
	Passenger			Freight		Passenger & Freight			
	Motorcycle	Car	Bus	LDV	HDV	Train	Boat	Ship	Plane
Petrol	✓	✓	✓	✓	✓				
Diesel		✓	✓	✓	✓	✓	✓	✓	
CNG			✓						
Electricity	✓	✓	✓	✓	✓	✓	✓		
Biofuel				✓	✓				✓
Fuel oil								✓	
Hydrogen				✓	✓			✓	
Ammonia								✓	
Jet fuel									✓

⁎ Note: Both international and domestic transport are included for maritime and aviation.

### 3.1 Population and GDP

**Fig. 1:**
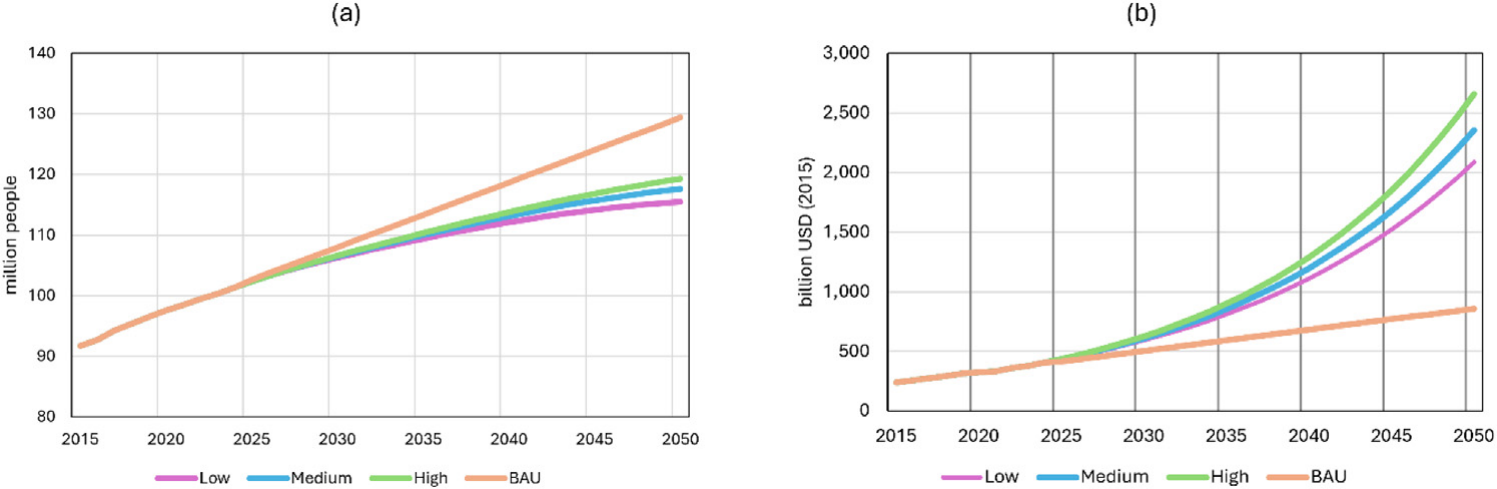
Historical (2015–2024) and forecasted (2025–2050) (a) population and (b) GDP at low, medium, and high rates for Vietnam.

Historical total population (millions) and annual population growth rate (%) for the period 2015 to 2024 were obtained directly from the yearly socio-economic database of the Vietnam NSO [3]. The specific source for each year's socio-economic database is noted in the repository. The NSO also provides population projections under low, medium, and high scenarios to 2049, through the Vietnam Population Projection 2014–2049 report [7]. Similarly, historical total GDP (constant 2015 billion USD) and annual GDP growth rate (%) for 2015 to 2024 were compiled from the World Bank DataBank [8]. Long-term GDP projections to 2050, under low, medium, and high scenarios, were derived from Vietnam’s Power Development Plan VIII (PDP8), which sets targets of “*an average GDP growth of about 7% per year during 2021–2030, and about 6.5–7.5% per year during 2031–2050″* [9]. The business-as-usual (BAU) scenario is also included and reflects an extrapolation of historical data. Fig. 1 illustrates population and GDP trends and forecasts.

### 3.2 Passenger activity

**Fig. 2:**
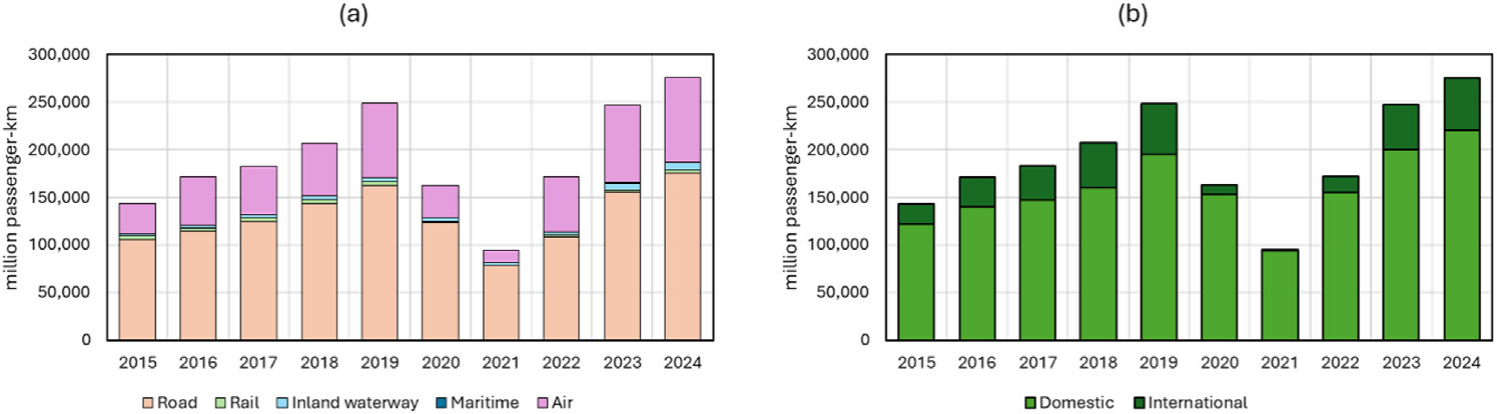
Historical passenger activity in Vietnam from 2015 to 2024 by (a) mode type and (b) travel type.

Passenger activity, measured in million passenger-km, was compiled for the transport modes defined in Table 2, for 2015 to 2024. These data were sourced directly from the annual socio-economic database of NSO [3]. The specific source for each year's socio-economic database is noted in the repository. In addition to total passenger activity by sector (road, rail, IWW, maritime, and air), the dataset also distinguishes travel type (domestic and international). Further calculations, described in Section 4.1, were applied to disaggregate passenger activity by vehicle and fuel type. Fig. 2 presents the resulting data, disaggregated by vehicle category and travel type.

### 3.3 Freight activity

**Fig. 3:**
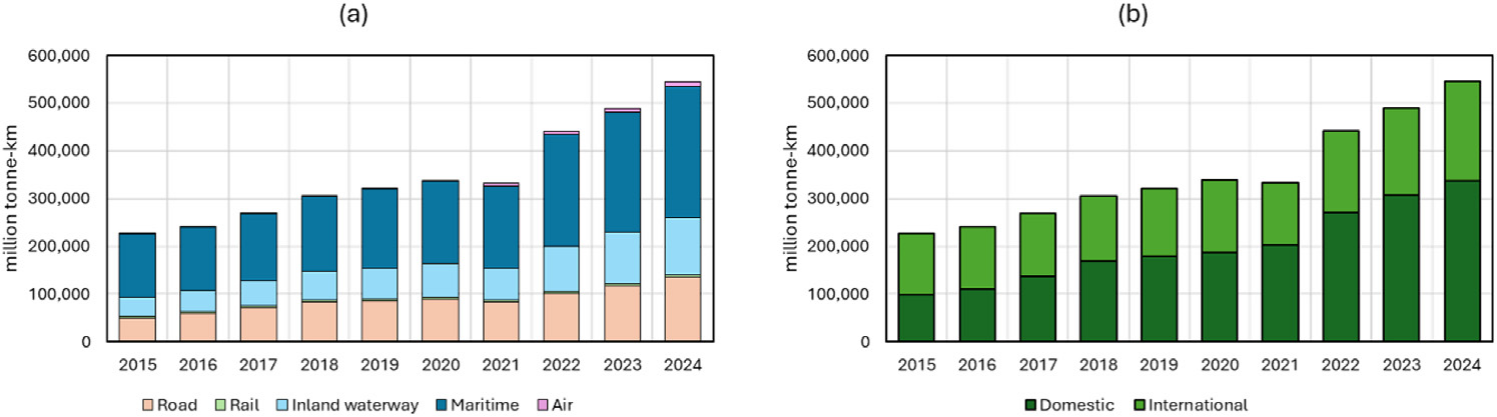
Historical freight activity in Vietnam from 2015 to 2024 by (a) mode type and (b) travel type.

Freight activity, measured in million tonne-km, was compiled for the transport modes defined in Table 2, for 2015 to 2024. These data were sourced directly from the annual socio-economic database of NSO, with the specific source for each year provided in the data repository. In addition to total passenger activity by sector (road, rail, IWW, maritime, and air), the dataset also distinguishes travel type (domestic and international). Further calculations, described in Section 4.1, were applied to disaggregate freight activity by vehicle and fuel type. Fig. 3 presents the resulting data, disaggregated by vehicle category and travel type.

### 3.4 Vehicle stock

**Fig. 4:**
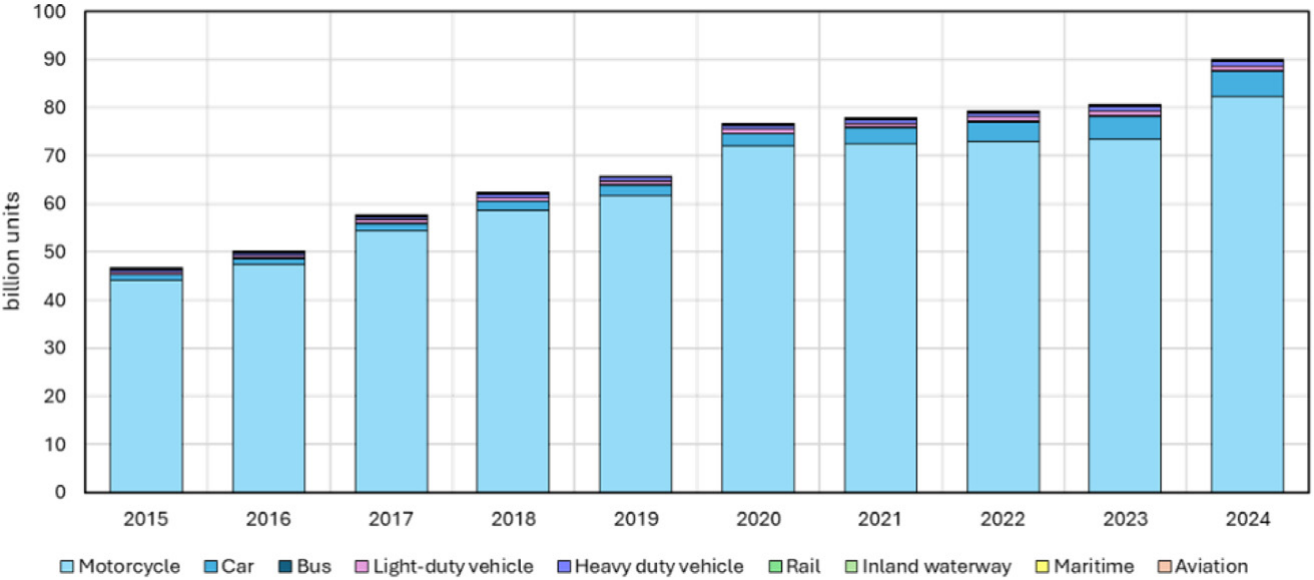
Historical transport vehicle stock in Vietnam from 2015 to 2024 by type.

Vehicle stock data was obtained from multiple sources, including the TLSY [2], the Ministry of Transport’s presentation on Decarbonising Transport Strategies in Vietnam [10], and the ASEANStats database [4]. Additional sources include local online news articles, which are noted in the data repository. Data for 2015 to 2024 were compiled and subsequently calibrated, as described in Section 4.2, to estimate vehicle stock by type and to further disaggregate them by fuel. Fig. 4 presents the total vehicle stock data.

### 3.5 Energy intensity

**Fig. 5:**
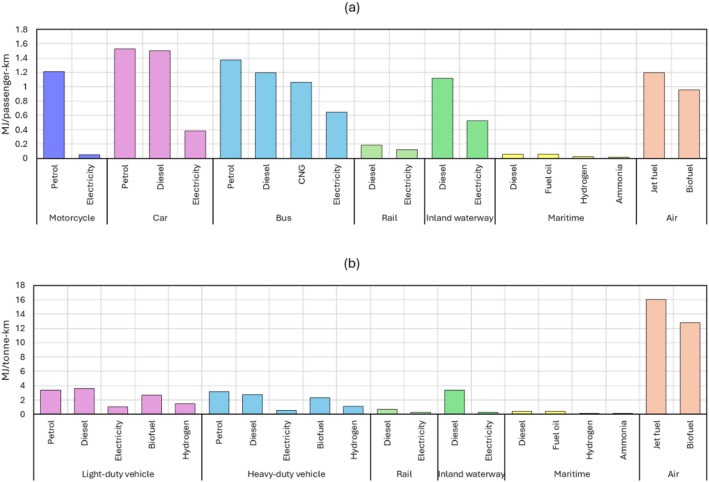
Energy intensity for each (a) passenger and (b) freight vehicle type, by fuel, in Vietnam.

Energy intensity, expressed in MJ/passenger-km and MJ/tonne-km, is provided for each transport vehicle by fuel type. Because specific vehicle-by-fuel data are challenging to obtain, most values were estimated using secondary sources such as online transport company specifications, institutional and academic databases, and peer-reviewed journals. The methodology used to calculate energy intensity for each mode–fuel type is described in Section 4.3. As a result, many of the values are not country or year-specific but are adapted from data available for other Asian and European countries. Nonetheless, Vietnam-specific estimates were derived for electric motorcycles and electric HDVs. Further, year-specific data were inaccessible; thus, the dataset presents energy intensity as a time-independent variable. Nonetheless, users can adopt, adapt, and apply these time-independent values in their analyses. Fig. 5 presents the resulting energy intensity values for each transport vehicle by fuel type.

### 3.6 Load capacity

Load capacity, expressed in passenger/vehicle and tonne/vehicle, is provided for each vehicle type. Since country-specific data are not readily available online, most of the values were estimated using general averages or data from other Asian countries. The load capacity data were sourced from academic literature, institutional reports, and media posts. Further, year-specific data were inaccessible, so load capacity is presented as a time-independent variable in the dataset. Nonetheless, users can adopt, adapt, and apply these values in their analyses. The estimation methods are detailed in Section 4.4. Table 3 notes the value for each vehicle type.

**Table 3:** Load capacity for each vehicle type.

Passenger		Freight	
Mode	Load capacity (passenger/vehicle)	Mode	Load capacity (tonne/vehicle)
Motorcycle	1.1	LDV	1
Car	1.7	HDV	5.5
Bus	19.8		
Rail	370	Rail	225
IWW	68.3	IWW	300
Maritime	107	Maritime	14,472
Air	172.5	Air	15.8

### 3.7 Carbon emission factors

**Fig. 6:**
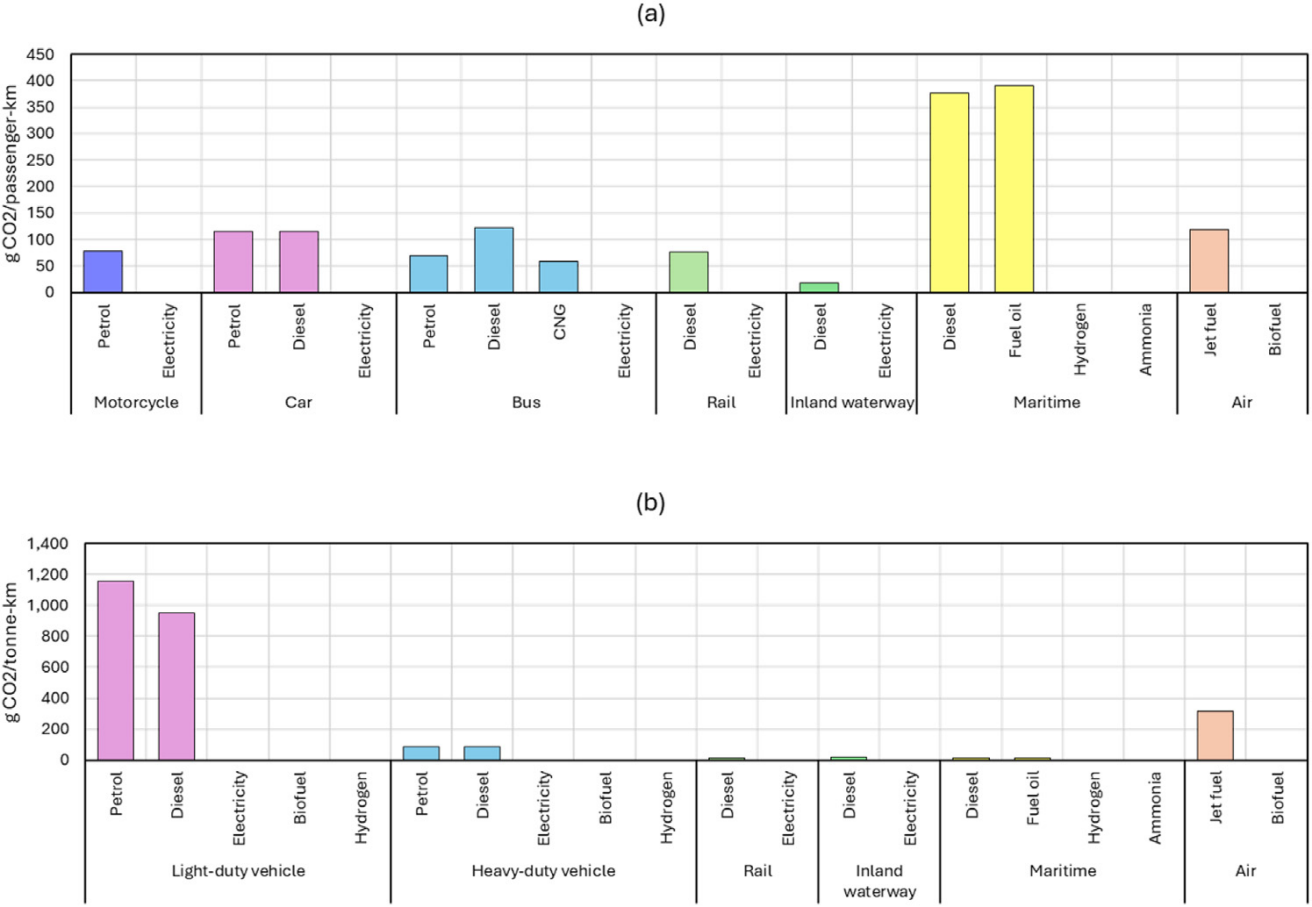
CO_2_ emission factors for each (a) passenger and (b) freight vehicle type, by fuel, in Vietnam.

CO_2_ emission factors, expressed in gCO_2_/passenger-km and gCO_2_/tonne-km, are included in the dataset. Only CO_2_ emissions are considered, as the primary objective is to model Vietnam’s net-zero carbon strategy. Thus, while NO_x_ and SO_x_ emissions are important for assessing local air quality, they are outside the scope and omitted from the dataset. Most values were derived from the Joint Research Centre’s Integrated Database of the European Energy System (IDEES) [11], which is primarily based on European data. While regional differences exist, European emission factors are considered a reasonable proxy for Vietnam due to similarities in the underlying vehicle technologies, fuel characteristics, and international reporting standards that guide emissions estimation [12]. Where emission factors were unavailable, additional values were calculated as detailed in Section 4.5. For clean technologies such as electricity, hydrogen, and ammonia, zero direct emissions are assumed. Biofuels are also treated as carbon-neutral based on sustainable local resource use, which is a standard assumption in energy systems modelling to simplify the complex life-cycle analysis of biomass [[13], [14], [15]]. It is important to note that this dataset's scope does not extend to upstream emissions. Year-specific data were also inaccessible, so the dataset presents carbon emission factors as a time-independent variable. Nonetheless, users can adopt, adapt, and apply these values in their analyses. Fig. 6 presents the CO_2_ emission factors by transport vehicle and fuel type.

## 4 Experimental Design, Materials and Methods

### 4.1 Transport activity

Data for road, rail, IWW, maritime, and air transport from 2015 to 2024, in passenger-km and tonne-km, were obtained from the NSO socio-economic database [3], with direct links to each year’s database provided in the repository. However, the NSO data are not disaggregated by fuel type, and road transport is not separated by vehicle category (motorcycle, car, bus, LDV, HDV). Thus, transport activity by vehicle and fuel type was estimated by disaggregating total activity according to the proportional shares of vehicle stock reported in the repository. To further separate domestic and international transport activity, road, rail, and IWW were assumed to be fully domestic, while maritime and aviation were treated as having domestic and international travel. This assumption follows JICA’s report [16], which highlights maritime and aviation as the primary modes for international travel in Vietnam. Domestic–international travel ratios for maritime and air were then calculated using (1), (2), and then applied to total activity to derive the respective shares. Additionally, data for maritime fuel use between diesel and fuel oil for both domestic and international travel was available from the 2018 TLSY [2]. These values were therefore used to assume the split of fuel use. However, there was no data for international passenger maritime transport, and so it was assumed that the split of fuel between fuel oil and diesel for international passenger maritime travel is the same as that for international freight maritime transport. Furthermore, since only a single test flight using sustainable aviation fuel (Singapore–Hanoi) was reported in 2024, we assume that all aviation fuel consumption is entirely jet fuel [17].

(1) $$\begin{array}{ccc} & & \text{Domestic}\;\text{travel}\;\text{ratio}\\ & & =\;\frac{\text{Domestic}\;\text{transport}\;\text{activity}\;\text{for}\;\text{all}\;\text{modes}-\text{Domestic}\;\text{transport}\;\text{activity}\;\text{for}\;\text{road},\;\text{rail},\;\text{and}\;\text{IWW}}{\text{Domestic}\;\text{and}\;\text{international}\;\text{transport}\;\text{activity}\;\text{for}\;\text{maritime}\;\text{and}\;\text{aviation}} \end{array}$$

(2) Internationaltravelratio=1−Domestictravelratio

### 4.2 Vehicle stock

Vehicle stock data from 2015 to 2024, where available, were obtained from multiple sources, including the TLSY [2], the Ministry of Transport’s presentation on Decarbonising Transport Strategies in Vietnam [10], and the ASEANStats database [4]. Additional sources include local online news articles, which are noted in Table 4. However, some discrepancies were observed across sources. For example, ASEANStats reported 44,128,000 motorcycles in 2015, while TLSY reposted 44,128,822. In this case, since all motorcycles were recorded as petrol in 2015, the average of the two values was used. Similarly, ASEANStats recorded 1033,000 car units in 2015, whereas TLSY reported 1033,131; however, the TLSY value did not align with the sum of petrol (992,936) and diesel (74,401) cars in the same dataset. In this case, as the missing remainder was not addressed in the TLSY dataset, the sum of the petrol and diesel cars (1067,637) was used for consistency. This methodology was applied to buses, LDVs, and HDVs, which operate on multiple fuel types. For these modes, vehicle stocks were aggregated by mode-fuel type, rather than averaged, to more accurately reflect the distribution of fuels within each mode category.

**Table 4:** Additional sources used for the estimation of vehicle stock.

Type	Mode	Fuel	Source	Source ID
Passenger	Bus	CNG	VietnamPlus, ‘Ho Chi Minh City works to extend CNG bus routes’, 2017. Available online.	A
			Southeast Asia Infrastructure, ‘Plans announced to expand CNG infrastructure in Ho Chi Minh City’, 2024. Available online.	B
			Hanoi Times, ‘Hanoi targets up to 90% electric and green energy buses by 2030′, 2024. Available online.	C
	Rail	Electricity	VietnamPlus, ‘Cat Linh - Ha Dong metro line welcomes 7.2 m passengers in a year of commercial operations’, 2022. Available online.	D
			Hanoi Times, ‘Hanoi's second metro line opens’, 2024. Available online.	E
Freight	Aviation	Jet fuel	Viet Nam Net Global, ‘With only 12% of air-cargo market share, Vietnam yields to regional countries’, 2023. Available online.	F

Electric vehicle stock was also estimated as the residual stock after subtracting the recorded petrol and/or diesel vehicle stocks from total reported vehicle numbers, based on literature noting electricity as a rapidly growing fuel type in Vietnam, behind petrol and diesel [18]. For freight rail and IWW, all stock was assumed to be diesel, as TLSY reported that freight rail is exclusively diesel-powered [2] and given the lack of data and evidence on electrification in these sectors. Further, no vehicle stock data were identified for 2024. Therefore, historical data were extrapolated to 2024. Similarly, gaps between 2015 and 2023 were filled by extrapolating from available historical data following observed trends. All extrapolations are documented in the repository, with accessible Excel formulas for transparency.

### 4.3 Energy intensity

Energy intensity values in MJ/passenger-km or MJ/ton-km were not readily available and, therefore, had to be derived for each vehicle-fuel type combination. For most vehicle-fuel, Eq. (3) was applied using energy consumption data (kgoe) from the IDEES [11], together with load capacity data from Table 3. Only CNG buses use Eq. (4). As three energy consumption values were available (source C), an average was calculated. The differences between these values were small (115 kg/km), so using the average was considered appropriate. For electric vehicles, Eq. (5) was used instead, based on battery capacity and distance-per-charge data obtained from specific vehicle manufacturers' sales pages (sources A, B, E, G, H, I). Where possible, locally relevant sources were prioritised. For example, data on electric motorcycles were collected from the local company VinFast (sources A, B). However, it should be noted that the distance-per-charge data reported by VinFast is based on standardised test conditions (e.g., 30km/hr, single rider of 65 kg). Thus, the estimated energy intensity may vary under different operating conditions.

Additionally, Hao et al. [19] reported that petrol-fueled vehicles consume approximately 15% more energy than comparable diesel-fueled vehicles, and that hydrogen-fueled vehicles consume about 40% of the diesel equivalent. These relationships were applied where direct data for petrol or hydrogen vehicles were unavailable. Where no direct or indirect estimates could be obtained, energy intensity values were approximated using proportional energy content ratios relative to diesel. This approach was applied to certain vehicle types operating on fuel oil, biofuel, and ammonia. Overall, due to a lack of Vietnam-specific energy intensity data for transport vehicles, placeholder values from other Asian and European regions were used. The additional sources used for estimating energy intensity by mode-fuel type are noted in Table 5.

(3) $$\begin{array}{ccc} & & \text{Energy}\;\text{intensity}\;\left(\frac{MJ}{pkm\;}\;\text{or}\;\frac{MJ}{tkm}\right)\\ & & \;=\;\text{Energy}\;\text{consumption}\;\left(\frac{kgoe}{100\;km}\times \frac{41.868\;MJ}{kgoe}÷100\right)÷\text{Load}\;\text{capacity}\;\left(p\;\text{or}\;t\right) \end{array}$$

(4) $$\text{Energy}\;\text{intensity}\;\left(\frac{MJ}{pkm\;}\right)=\text{Energy}\;\text{consumption}\;\left(\frac{kg}{km}\times \frac{MJ}{kg}\right)÷\text{Load}\;\text{capacity}\;\left(p\right)$$

(5) $$\begin{array}{ccc} \text{Energy}\;\text{intensity}\;\left(\frac{MJ}{pkm}\text{or}\frac{MJ}{tkm}\right) & = & \text{Battery}\;\text{capacity}\;\left(kWh\;\times \frac{3.6\;MJ}{kWh}\right)\\ & & ÷\;\text{Distance}\;\text{per}\;\text{charge}\;\left(km\right)÷\text{Load}\;\text{capacity}\;\left(p\;\text{or}\;t\right) \end{array}$$

**Table 5:** Additional sources used for estimating energy intensity by mode-fuel type.

Type	Mode	Fuel	Source	Source ID
Passenger	Motorcycle	Electricity	VinFast, ‘Evo Lite Neo’, 2025. Available online.	A
			VinFast, ‘Evo 200′, 2025. Available online.	B
	Bus	CNG	Prati, M.V., Costagliola, M.A., Unich, A., Mariani, A., ‘Emission factors and fuel consumption of CNG buses in real driving conditions’, Transportation Research Part D: Transport and Environment, vol. 113, p. 103,534, 2022	C
	IWW	Diesel	Newcombe, K., ‘Energy use in Hong Kong: Part II, sector end-use analysis’, Urban Ecology, 1(2–3), 285–309, 1975.	D
		Electricity	Ampereship, ‘Type ASP-300′, 2025. Available online.	E
	Maritime	Diesel	Teske, S., & Niklas, S., ‘Decarbonisation pathways for transport’. In Achieving the Paris Climate Agreement Goals: Part 2: Science-based Target Setting for the Finance industry—Net-Zero Sectoral 1.5° C Pathways for Real Economy Sectors, pp. 187–222. Cham: Springer International Publishing, 2022.	F
Freight	HDV	Electricity	BYD, ‘ETM 6′, n.d. Available online.	G
			BYD, ‘ETH 8′, n.d. Available online.	H
	IWW	Electricity	Elektrek, ‘Check out the Netherlands' first electric inland ship - and it's got swappable batteries’, 2021. Available online.	I

### 4.4 Load capacity

Load capacity data, in passenger/vehicle or load/vehicle, were collected and derived from various sources, including academic journals, institutional reports, and media articles, with exact references provided in Table 6.

**Table 6:** Sources used for the collection of load capacity data.

Type	Mode	Source	Source ID
Passenger	Motorcycle	Hung, D.V., Stevenson, M.R., Ivers, R.Q., ‘Barriers to, and factors associated with, observed motorcycle helmet use in Vietnam’, Accident Analysis & Prevention, vol. 40, no. 4, pp. 1627–1633, Jul. 2008.	J
	Car	Fwa, T.F., Chua, G.K., ‘Passenger car travel characteristics in Singapore: Analysis of changes from 1990 to 2005′, IATSS Research, vol. 31, no. 2, pp. 48–55, 2007.	K
	Bus	Bakar, M.F.A., Ismail, N., Norhisham, S., Ahad, N.A., Okwonu, F.Z., Nor, S.E.M., Dullah, H., ‘Assessing the passenger load of urban bus performance in the eastern region of Peninsular Malaysia’, in Advances in Civil Engineering Materials: Selected Articles from the 6th International Conference on Architecture and Civil Engineering (ICACE 2022), Springer Nature Singapore, Singapore, 2023, pp. 323–332.	L
	Rail	Logistics Cluster, ‘Viet Nam railway assessment’, Logistics Capacity Assessment, 2024. Available online.	M
	IWW	Utomo, D.M., Mateo-Babiano, I., ‘Exploring indigeneity of inland waterway transport (IWT) in Asia: Case studies of Thailand, Vietnam, the Philippines, and Indonesia’, Journal of the Eastern Asia Society for Transportation Studies, vol. 11, pp. 2316–2332, 2015.	N
	Maritime	Lee, S., Ramdeen, C., ‘Cruise ship itineraries and occupancy rates’, Tourism Management, vol. 34, pp. 236–237, 2013.	O
	Air	IEA-ETSAP, ‘Aviation transport’, Energy Technology Data Sheet T12, International Energy Agency – Energy Technology Systems Analysis Programme, 2011. Available online.	P
Freight	LDV and HDV	Agora Verkehrswende, ‘Towards decarbonising transport in Vietnam’, Agora Verkehrswende, Berlin, 2024. Available online.	Q
	IWW	World Bank, ‘Sustainable development of inland waterways transport in Vietnam: Strengthening the regulatory, institutional and funding frameworks’, World Bank, Washington, DC, 2019. Available online.	R
	Maritime	Statista, ‘Average cargo carrying capacity per vessel in Vietnam in 2021, by type’, Statista Research Department, 2021. Available online.	S
	Air	Reed Smith, ‘Carrying the load: The use of passenger aircraft to haul cargo during the COVID-19 pandemic’, Reed Smith LLP, 2022. Available online.	T

For certain modes, such as motorcycles and aviation, load capacities were estimated from reported sample data. For motorcycles, source J reported a sample of 716 motorcycle drivers and 92 motorcycle passengers. Thus, load capacity was calculated by dividing the total number of drivers and passengers by the number of drivers. For aviation, source P reported load capacities of 80% for a 150-seat aircraft and 75% for a 300-seat aircraft. These values were first weighed by aircraft size and then averaged. For bus and freight maritime transport, multiple values from sources L and S were available, and they were averaged to derive a representative load capacity. For modes such as cars and rail transport, load capacity values were taken directly from the sources K and M without further modification. Where data were incomplete or unclear, informed assumptions were applied based on available evidence. For example, source Q noted a policy target load factor of 60% for LDVs by 2030. On this basis, a conservative load factor of 50% was assumed for the 2020s, applied to a vehicle capacity of 2 tonnes. Where available, country-specific data were used (source A, D, H, K, L). However, when Vietnam-specific information was unavailable, placeholder values derived from comparable Asian contexts were used.

### 4.5 Carbon emission factors

CO_2_ emission factors, in gCO_2_/passenger-km and gCO_2_/tonne-km, were obtained directly from the IDEES [11] for most vehicle-fuel combination types. While these values are based on European data, they were assumed to apply to Vietnam due to similarities in the underlying vehicle technologies, fuel characteristics, and international reporting standards that guide emissions estimation [12]. As there is no national emission inventory, these proxy values offer a transparent interim basis for analysis. Additional data for CNG buses, IWW and maritime fuel oil emission factors were sourced from other academic and institutional references, which are documented in Table 7. For CNG buses, source C provided three values, and the average was used. Where no data were available, emission factors were estimated based on the relative emission content of each fuel compared to diesel. This approach was applied to diesel maritime and petrol HDVs. In this case, the fuel-specific emission content values for petrol, diesel, and fuel oil were first obtained (Table 8) and expressed as ratios relative to diesel. These ratios were then applied to the corresponding diesel emission factors for the relevant transport modes, using diesel-equivalent values sourced from the IDEES [11].

**Table 7:** Additional sources used for the collection of CO_2_ emission factors.

Type	Mode	Fuel	Source	Source ID
Passenger	Bus	CNG	Prati, M.V., Costagliola, M.A., Unich, A., Mariani, A., ‘Emission factors and fuel consumption of CNG buses in real driving conditions’, Transportation Research Part D: Transport and Environment, vol. 113, p. 103,534, 2022	C
	IWW	Diesel	Department for Energy Security and Net Zero, ‘Greenhouse gas reporting: Conversion factors 2024′, UK Government, 2024. Available online.	U
	Maritime	Fuel oil	Howitt, O.J., Revol, V.G., Smith, I.J., Rodger, C.J., ‘Carbon emissions from international cruise ship passengers’ travel to and from New Zealand’, Energy Policy, vol. 38, no. 5, pp. 2552–2560, 2010	V

**Table 8:** Emission content by fuel for petrol, diesel, and fuel oil.

Fuel	gCO_2_/MJ	Source
Petrol	69.96	SEAI, ‘Conversion factors’, n.d. Available online.
Diesel	73.30	
Fuel oil	76.01	

## Limitations

This dataset was compiled through desk-based data collection from existing open-access sources, with limited access to primary data. Some inputs originate from manufacturers and may reflect commercially influenced assumptions, introducing potential bias. For example, electric motorcycle energy intensity was calculated from distance-per-charge data reported by VinFast, which is based on standardised test conditions (e.g., 30km/hr, single rider of 65 kg). Thus, the estimated energy intensity may vary under different operating conditions. Where data gaps occurred, assumptions were made using averages from available data, proportional calculations based on similar contexts, or extrapolations from historical trends. In some cases, proxy data from other countries in the Asian region were used to complement missing national information. For specific parameters such as energy intensity and CO_2_ emission factors, data were adopted from European sources due to their availability. As a result, there is potential for bias, since these assumptions may not fully capture the evolving characteristics of Vietnam’s transport sector. Furthermore, the limited granularity of some sources means that certain vehicle types, activity levels, and energy use are represented by broad averages, which may reduce accuracy. These limitations should be considered when applying the dataset for detailed modelling or policy analysis.

## Ethics Statement

The authors have read and followed the ethical requirements for publication in Data in Brief and confirm that the current work does not involve human subjects, animal experiments, or data collected from social media platforms.

## CRediT Author Statement

**Naomi Tan:** Conceptualisation, Methodology, Validation, Data Curation, Writing – Original Review & Editing, Visualisation. **Fynn Kiley:** Validation, Writing - Review & Editing. **Holger Dalkmann:** Writing - Review & Editing, Supervision. **Johanna Zilliacus:** Writing - Review & Editing. **John Harrison:** Writing - Review & Editing, Supervision. **Mark Howells:** Supervision**. Vivien Foster:** Supervision.

## Data Availability

ZenodoDataset to Support the Modelling of Vietnam's Transport Sector: Population, Economy, Transport Activity, Energy Efficiency, Load Capacity, and Carbon Emission (Reference data)
